# Effects of SGLT2 Inhibitors and GLP-1 Receptor Agonists on Renin-Angiotensin-Aldosterone System

**DOI:** 10.3389/fendo.2021.738848

**Published:** 2021-10-21

**Authors:** Soraya Puglisi, Alessandro Rossini, Roberta Poli, Francesca Dughera, Anna Pia, Massimo Terzolo, Giuseppe Reimondo

**Affiliations:** ^1^ Internal Medicine, Department of Clinical and Biological Sciences, San Luigi Gonzaga Hospital, University of Turin, Orbassano, Italy; ^2^ Endocrinology and Diabetes Unit, ASST Papa Giovanni XXIII, Bergamo, Italy; ^3^ Metabolic Disease and Diabetes Unit, San Luigi Gonzaga Hospital, Orbassano, Italy

**Keywords:** diabetes mellitus type 2, cardiovascular disease, cardiovascular risk, diabetic kidney disease, sodium-glucose cotransporter-2 inhibitor, glucagon-like peptide-1 receptor agonist, aldosterone, renin

## Abstract

Sodium-glucose cotransporters inhibitors (SGLT2-i) and GLP-1 receptor agonists (GLP1-RA) are glucose-lowering drugs that are proved to reduce the cardiovascular (CV) risk in type 2 diabetes mellitus (T2DM). In this process, the renin-angiotensin-aldosterone system (RAAS) is assumed to play a role. The inhibition of SGLT2 improves hyperglycemia hampering urinary reabsorption of glucose and inducing glycosuria. This “hybrid” diuretic effect, which couples natriuresis with osmotic diuresis, potentially leads to systemic RAAS activation. However, the association between SGLT2-i and systemic RAAS activation is not straightforward. Available data indicate that SGLT2-i cause plasma renin activity (PRA) increase in the early phase of treatment, while PRA and aldosterone levels remain unchanged in chronic treated patients. Furthermore, emerging studies provide evidence that SGLT2-i might have an interfering effect on aldosterone/renin ratio (ARR) in patients with T2DM, due to their diuretic and sympathoinhibition effects. The cardio- and reno-protective effects of GLP-1-RA are at least in part related to the interaction with RAAS. In particular, GLP1-RA counteract the action of angiotensin II (ANG II) inhibiting its synthesis, increasing the inactivation of its circulating form and contrasting its action on target tissue like glomerular endothelial cells and cardiomyocytes. Furthermore, GLP1-RA stimulate natriuresis inhibiting Na+/H+ exchanger NHE-3, which is conversely activated by ANG II. Moreover, GLP1 infusion acutely reduces circulating aldosterone, but this effect does not seem to be chronically maintained in patients treated with GLP1-RA. In conclusion, both SGLT2-i and GLP1-RA seem to have several effects on RAAS, though additional studies are needed to clarify this relationship.

## Introduction

Cardiovascular disease (CVD) is the main cause of morbidity and mortality in patients with type 2 diabetes (T2DM), with a relevant impact on the economic costs for health care systems and on the quality of life for many patients (1, 2). Due to the increasing epidemic boost of T2DM in recent years, whose trend will be confirmed in the next decades (3, 4), the need of reducing the CV risk in diabetic patients has become a priority. Therefore, the research has focused on new drugs that have been showed to have pleiotropic effects beyond hypoglycemic action.

Among the novel therapies, great interest has been aroused by sodium–glucose cotransporter-2 inhibitors (SGLT2-i) and glucagon-like peptide-1 receptor agonist (GLP1-RA), that have changed the management of diabetic patients. Indeed, the experience of several randomized control trials has been included in the most recent guidelines (5, 6), which recommended these classes of drugs for patients with T2DM and CVD.

In patients with T2DM, the treatment with SGLT2-i has been associated with a reduction in hospitalization for heart failure and cardiovascular deaths, regardless of pre-existing cardiovascular disease (7–11). Moreover, among patients with T2DM and established cardiovascular disease, some trials reported the additional benefit of a lower rate of major adverse cardiac events (MACE) in patients treated with SGLT2-i (7, 10).

Several studies (12–15) have demonstrated that GLP1-RA treatment variably reduced MACE incidence, mainly in T2DM patients with established cardiovascular disease or multiple cardiovascular risk factors, although only one of them (12) showed a benefit on mortality risk. Conversely, the positive effects of SGLT2-i on hospitalization for heart failure has not been reported for GLP1-RA.

This heterogeneity in CV benefits across the trials, also among studies in which drugs of the same class have been used, could be explained by different study designs (i.e. inclusion criteria, follow-up period) (16), but it could be also due to peculiar and still unknown effects of each drug.

Indeed, the underlying mechanisms of these drugs on the CV system are not definitively understood. It has been suggested that the endocrine system could play a role, especially the renin-angiotensin-aldosterone system (RAAS). This review aims to summarize the available evidence on this topic.

## The Renin-Angiotensin-Aldosterone System (RAAS)

It is well known that the RAAS is one of the most important regulators of arterial blood pressure, with a key role in cardiovascular and renal diseases, mainly mediated by inflammatory processes (17, 18). Although renin was discovered more than a century ago and the detection of angiotensin I, angiotensin II and aldosterone in the ‘50s allowed the definition of the classical RAAS pathway from 1961 (19), the research in this field continues and new evidence has recently emerged.

The cascade of the systemic RAAS starts with the liver angiotensinogen (AGT), which is converted to Angiotensin I (ANG I) by renin, a hormone secreted by the juxtaglomerular cells. The angiotensin-converting enzyme (ACE), secreted by lung and kidney, converts ANG I to ANG II, whereas the angiotensin-converting enzyme 2 (ACE2) further cleaves ANG II to ANG (1,7). The balance between ACE and ACE2 activity influences the ultimate effect of RAAS, since ANG II promotes vasoconstriction and proliferation while ANG (1,7) stimulates vasodilation and apoptosis (20). ANG II acts through two receptor subtypes, the AT1R and the AT2R (21). While AT1R mediates classic ANG II actions such as vasoconstriction, aldosterone release, and sodium and water retention, ANG II signaling through AT2R has opposite effects and may represent a protective mechanism against an overstimulation of AT1R (21).

Recently, three new axes have been identified, emphasizing the role of the kidney in this picture (22):

1. The ACE2/ANG(1-7)/Mas receptor pathway (23, 24): Ang-(1-7) binds to the G protein-coupled receptor Mas, which is expressed in renal proximal tubular cells, afferent arterioles, cardiac myocytes, and neuronal cells; this pathways is responsible of the ANG(1-7) vasodilatory effects, through the release of bradykinin, prostaglandin and endothelium-dependent nitric oxide.
2. The prorenin/PRR/MAP kinases ERK1/2 axis: in the past years, renin was considered simply as an enzyme catalyzing the conversion of AGT into ANG I. However, recent studies have highlighted that prorenin and/or active renin can bind to prorenin receptor (PRR), which is expressed in glomerular mesangial cells, collecting ducts, and the subendothelium of renal arteries. The binding of prorenin to PRR triggers the phosphorylation of mitogen-activated protein kinases/extracellular regulated kinases 1/2 (MAPK/ERK1/2), which is demonstrated to be an ANG II-independent mechanism involved in the development of diabetic nephropathy (25)
3. The ANGIV/AT4/IRAP cascade: ANG IV (derived from the metabolization of ANG II through aminopeptidases A and N) binds the AT4 receptor, which has been identified as the insulin-regulated aminopeptidase (IRAP), but the role of this pathway in the blood pressure and renal regulation is still uncertain (22).

These new axes have raised great interest because their targeting could provide novel therapeutic strategies for hypertension, cardiovascular and kidney diseases.

Similarly, the discovery of a RAAS chronobiology could represent a milestone to change the paradigm of treatment of these pathologies. The concept of the circadian rhythmicity in the human secretion of renin and aldosterone, under the influence of posture, sodium intake and age, is well known from decades (26). More recently, Mochel et al. have demonstrated that sodium intake interacted with the tonic and the phasic secretion of renin in dogs, suggesting that variations in feeding time could impact the chronobiology of the RAAS and blood pressure (27, 28). Moreover, Mochel et al. postulated that the optimal efficacy of drug affecting RAAS (for example ACE inhibitors) should be expected with bedtime dosing (29). This hypothesis has been confirmed in clinical trials including hypertensive patients that demonstrated an improved blood pressure control (30, 31) and reduction of cardiovascular events (31) when anti-hypertensive drugs have been administered at bedtime.

## Hyperglycemia and RAAS in the Kidney

High glucose levels in the proximal tubule can cause increased reabsorption of glucose, sodium and water, favored by the upregulation of SGLT2 activity and expression of Na-K-2Cl cotransporter, acquaporin-2, and urea transporters, as reported in diabetic patients (32). The resulting decrease in distal sodium chloride delivery promotes renal hemodynamic dysfunction by impairing the tubulo-glomerular feedback (TGF). This distal tubular condition is perceived as a low effective circulating volume stimulus, at the level of juxtaglomerular apparatus, which promotes the inhibition of adenosine production that is associated with a vasodilatory response of the afferent arteriole (33). The concomitant RAAS activation, which increases ANG II levels, causes efferent arteriolar vasoconstriction with consequent increase in renal perfusion and glomerular hyperfiltration. Noteworthy, hyperglycemia induces the synthesis of AT1R, which plays a pivotal role in reno- and cardiovascular disease. AT1R expression in mesangial cells and podocytes, induced by high glucose levels, promotes intracellular expression of profibrotic and pro-inflammatory mediators, such as transforming-growth factor beta (TGF- β), vascular endothelial growth factor (VGEF), and interleukin-6 (IL-6) leading to hyperplasia and hypertrophy, mainly of the proximal tubule, together with extracellular matrix production (34).

Accumulated evidence has shown that the RAAS is no longer considered to act just as an endocrine system, but also as a paracrine, autocrine and intracrine system. At tissue level, RAS (renin-angiotensin system) can be upregulated with non-hemodynamic effects (35).

Several studies reported that inappropriate ANGII/AT1R activation occurs because of different mechanisms, particularly in patients with T2DM. Firstly, hyperglycemia promotes local ANG II production in cardiomyocytes stimulating intracellular chymase and/or internalized prorenin, leading to diabetic cardiomyopathy. Secondly, high glucose concentrations have been shown to enhance the tissue response to ANG II. The third mechanism whereby diabetes upregulates ANGII/AT1R is ACE2 downregulation, resulting in local ANG (1-7) reduction and, consequently, in an imbalance of the RAS.

Lastly, diabetic patients have a high prorenin levels that could contribute more significantly than renin to the pathogenesis of end-organ damage by stimulating PRR intracellular signaling (35).

Also at kidney level, RAS compartmentalization and independent regulation have been shown to play an important role in the pathogenesis of hypertension and diabetic nephropathy (36). Indeed, it has been recognized that inappropriate activation of intrarenal RAS prevents the kidney from keeping normal Na^+^ balance at normal renal perfusion pressures together with promoting glomerular, tubular and interstitial inflammation and fibrosis.

Additional evidence for an independent intrarenal RAS included observations that intrarenal ANG II contents markedly increased compared to circulating levels. Furthermore, the intrarenal RAS can autoamplify ANG II production resulting in continuously intrarenal activation. In fact, the inter-relationship of AT1Rs to the intrarenal ANG II content is based on a feed-forward mechanism by which ATR1 binding mediates ANG II intracellular accumulation (37).

The reasons for elevated renal ANG II levels have been debated for years. AGT is present in the proximal tubule and concurrent increases in AGT-mRNA/protein in renal tissue homogenates has been explained as indicating that transcriptional activation of the AGT gene would be responsible for renal ANG II increase. However, more recent studies have demonstrated that the hepatic biosynthesis of AGT is the major source of renal ANG II concentrations and its renal expression depends on the integrity of the glomerular sieving function (38).

In fact, when the glomerular capillary wall, acting as a molecular barrier, is impaired, AGT protein is leaked into the tubular lumen and then reabsorbed at S1, S2 and S3 segments of proximal convoluted tubules (38). Matsutsaka et al. observed, in a murine model of selective podocyte damage, that massive filtration of liver-derived AGT led to enhanced renal ANG II content although renal renin was suppressed (39). Furthermore, augmented intraglomerular pressure induced by increased ANG II concentrations can promote further podocytes damage ensuring the formation of a feedback mechanism for ANG II synthesis, which is one of the key aspects of progressive glomerular disease (38).

The AGT reabsorption is a megalin-dependent process. Megalin, also known as low-density lipoprotein-related 2, is a multiligand endocytic receptor in the low-density lipoprotein family (LDL). Megalin is expressed along the apical aspect of proximal tubules, where it is responsible for hepatocyte-derived AGT reabsorption and its homeostasis in kidney. A recent study (40) demonstrated that inhibition of megalin, by antisense oligonucleotides, diminished both AGT and renin intra-tubular accumulation, while leading to increases of urine AGT and renin levels. Noteworthy, renal ANG II concentration decreased without affecting plasma ANG II concentrations. In addition to a regulatory role in intrarenal RAS homeostasis, megalin seems also to indirectly contribute to atherosclerosis development increasing renal ANG II levels (40).

## The Emerging Role of SGLT2-i in Cardio-Nephroprotection

SGLT2 is a low-affinity high-capacity co-transporter distributed mainly along the early segments of the proximal renal tubule. It is responsible for up to 97% of glucose renal reabsorption and for about 5% of total renal Na^+^ reabsorption.

**Figure 1:**
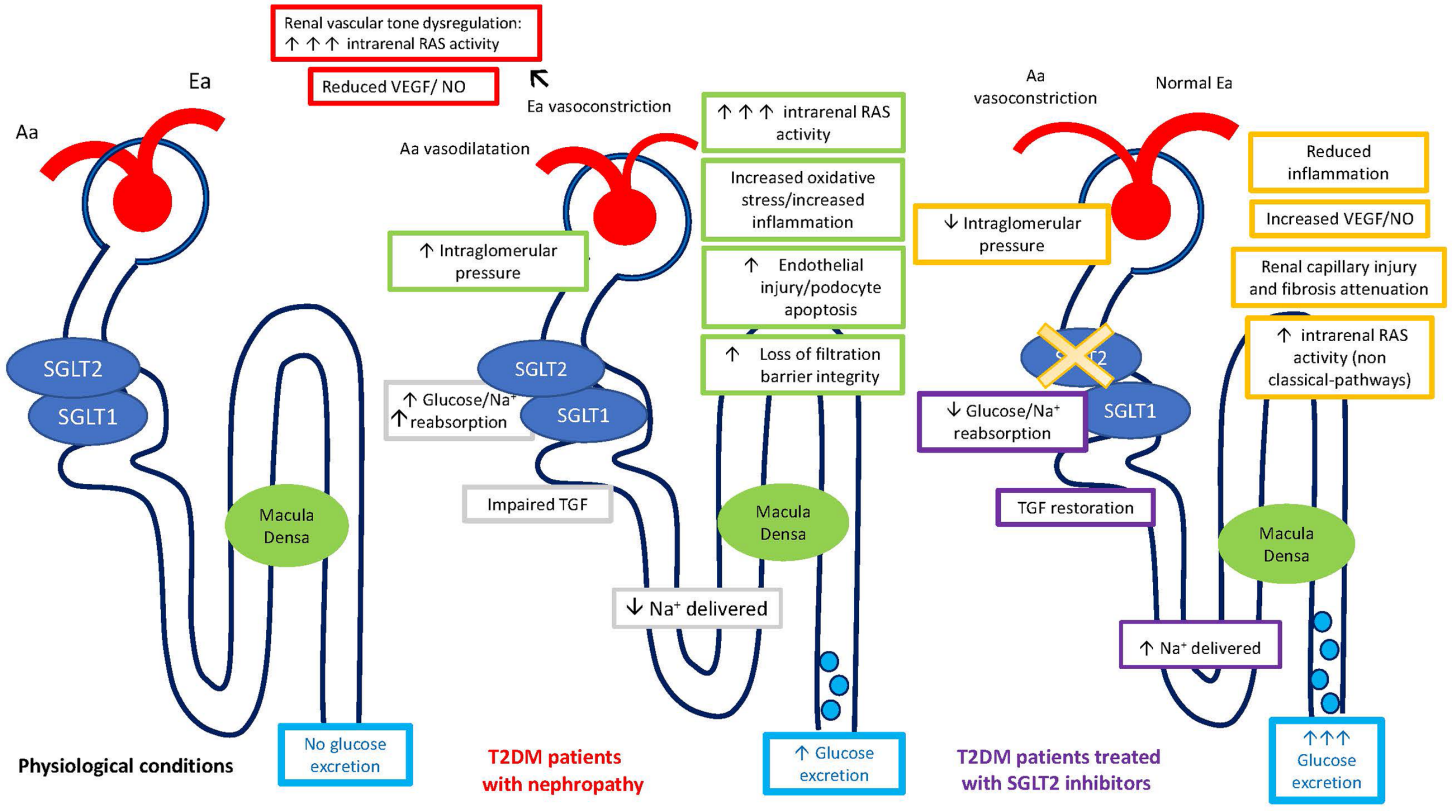
Regulation of single nephron glomerular filtration rate (SNGFR) by the tubular-glomerular feedback (TGF) and intrarenal RAS regulation in physiological conditions, in patients with diabetic nephropathy, and in diabetic patients treated with SGLT2-inhibitors. Aa, Afferent arteriole; Ea, Efferent arteriole; SGLT, Sodium-glucose cotransporters; T2DM, Diabetes Mellitus Type 2.

SGLT2-i are a relatively new class of antidiabetic drugs with an emerging role in cardio-nephroprotection. They improve glycemic control by inducing glycosuria, osmotic diuresis, and reduced glucose-sodium proximal tubule reabsorption. Inhibition of SGLT2 reduces sodium reabsorption in the proximal tubule and enhances Na^+^ delivery to the macula densa, promoting finally the TGF activation with consequent afferent arteriole vasoconstriction and reduction of the single-nephron-glomerular-filtration-rate (**Figure 1**). Attenuation of glomerular hyperfiltration results in prevention of long-term glomerular damage, contributing to reduce the progression of diabetic kidney disease (DKD) (41). Noteworthy, SGLT2-i have been shown to interfere with sodium-hydrogen exchanger (NHE-3), which is primarily responsible for sodium tubular reuptake following filtration and is markedly increased in heart failure (HF) (42).

The increased sodium delivery to the macula densa theoretically results in low renin levels *per se.* It has been suggested that SGLT2-i could exert a RAS-inhibition-like effect, without being associated with a real RAS-suppression. In fact, the more significant hemodynamic effects of this agents, conveyed by TGF restoration, led to afferent arteriole vasoconstriction as compared to efferent vasodilation observed with RAS-inhibition (43). The same authors reported an increase in all renal RAS markers under empagliflozin treatment during clamped euglycemia and hyperglycemia, reflecting a modest RAS activation because of osmotic diuresis. Moreover, the increased renal RAS activity might have been important in maintaining a normal glomerular filtration in this setting.

Pre-clinical studies in genetically modified animal model of T2DM (OLETF rats, Otsuka Long Evans Tokushima fatty rats) have shown high PRA (plasmatic renin activity) levels, without any change in aldosterone levels under dapagliflozin chronic treatment, in accordance with the aforementioned observations of an independent intrarenal RAS (36). Similarly, available clinical data have shown that PRA levels significantly increased after one month of treatment with SGLT2-i, while plasma aldosterone levels did not (44). Noteworthy, neither PRA nor plasma aldosterone significantly changed after six months of treatment, confirming the transient diuretic effect of SGLT2-i. Given the reabsorption of renin by proximal tubule cells through megalin, and the broad expression of ACE within the kidney, allowing locally synthetized ANG II independent action, the interference of SGLT-2-i with intrarenal RAS could explain such results.

Moreover, in recent years the use of small interfering RNAs (siRNA), selectively targeting AGT, as a novel approach of interfering with the RAS has shown beneficial cardio- and nephroprotective properties. Indeed, N-acetylgalactosamine-conjugated siRNA targeting AGT was highly effective in suppressing circulating AGT, thereby reducing blood pressure as ACE-inhibitors (ACE-i) and angiotensin-II receptor blockers (ARBs) (45). A study conducted by Uijl E. et al. on hypertensive mice highlighted that siRNA, especially in combination with valsartan, suppressed circulating and renal ANG II, resulting in renin levels increase, without affecting aldosterone levels. A concurrent decrease in NHE-3 has also been observed (46). Combining the AGT targeting siRNA with an ARB led to a stronger hypotensive effect likely because of the concomitant AT2R stimulation. However, the resulting complete ANG suppression II lowered proteinuria and cardiac hypertrophy, without a further improvement of glomerulosclerosis. This observation suggested that the complete inhibition of renal RAS could be detrimental (46).

Similarly, a possible SGLT2- i interfering effect on AGT/ANG II system together with intrarenal ANG II/AT2R pathways, especially when combined to RAAS blockers, can be argued.

In experimental models, OLETF rats have shown local RAS activation, which resulted in oxidative stress and kidney progressive damage, whereas treatment with dapagliflozin remarkably lowered the urinary ANG II and AGT levels (36). In contrast, Yoshimoto et al. reported that treatment with SGLT2-i did not affect the urinary AGT/creatinine ratio in T2DM patients (47). Taken together, this evidence suggests that the heterogeneity of studies models and the interaction of many adaptive mechanisms might explain the variable results in the recent studies concerning the influence of SGLT2-i treatment on the RAAS, although an activation of intrarenal RAS in chronic treated patients has been excluded (42).

However, the renal beneficial effect of SGLT2-i has been definitely reported in recent cardiovascular outcome clinical trials and further confirmed in CREDENCE, DAPA-CKD, EMPA-KIDNEY, which investigated renal outcomes as primary endpoints also in non-diabetic patients with chronic kidney disease (48). The potential main mechanism responsible for SGLT2-i reno-protective properties probably resides in TGF activation and glomerular hyperfiltration reduction. However, SGLT2-i could reduce renal and extra-renal glucotoxicity by inhibiting many pro-inflammatory pathways such as low serum ketone levels, hyperuricemia, oxidative stress and advanced glycation-end products (AGEs) induced by hyperglycemia (33). Furthermore, SGLT2-i interference with either generation or effect of ANG II at renal tissues site possibly plays a significant role in this setting.

Indeed, there is a growing body of evidence suggesting a significant beneficial class effect associated with SGLT2 inhibition, resulting in MACE, cardiovascular death and hospitalization for heart failure (HHF) reduction, despite some variabilities in the findings provided by individual CVOTs (40).

In populations that largely did not suffer from HF at the time of the enrolment, treatment with empagliflozin, canagliflozin and dapagliflozin had been able to decrease the risk of new-onset HF events by about 30%. Furthermore, in the EMPA-REG OUTCOME trial, empagliflozin reduced the risk of both pump failure and sudden deaths (46).

In addition, in the EMPEROR-reduced trial, enrolling about 3700 patients affected by heart failure with reduced ejection fraction (HFrEF), empagliflozin significantly reduced the risk of heart failure hospitalization and decreased total hospitalization for HF regardless of the presence of diabetes or baseline levels of glycohemoglobin (HbA1c) (49, 50).

Since the SGLT2-i benefits on HF could not be explained by anti-hyperglycemic action in any of these trials, multiple hypotheses have been raised about the potential mechanisms underlying their cardiovascular favorable effects.

It has been suggested that these drugs could act through the non-classic RAAS pathways in the context of simultaneous RAAS blockade. In fact, in the aforementioned trials most patients received appropriate treatment for HF, including ACE-i or ARBs. Therefore, the SGLT2-i induced elevated ANG II levels could act through AT2R resulting in vasodilation, sodium excretion, antiproliferative and anti-inflammation effects (51).

In addition, it has also been observed a SGLT2-i induced ACE2/ANG(1-7) pathway activation that may play a role in cardioprotection by competing with ANG II and AT1R (14). Both heart and kidney are major sources of ANG (1-7), which has broad effects in cardiovascular system, including vasodilation, myocardial protection, antiarrhythmic, antihypertensive, and positive inotropic effects (52).

Furthermore, the SGLT2-i “hybrid” diuretic effect, which combines natriuresis and osmotic diuresis, might also have cardioprotective properties leading to preload and myocardial stretch reduction (“the diuretic hypothesis”) (51). This effect has been observed especially in the early phase of treatment. In fact, SGLT2-i induced osmotic diuresis and reduction in plasma volume potentially activate systemic RAAS, leading to an initial increase of renin levels in the first three-six months of treatment (53). Conversely, in chronic treated patients the effects on volume changes are slightly reduced because a new steady state is reached, due to a counter-regulatory effect, resulting in a lower total body sodium concentration and blood volume (53).

Therefore, SGLT2-i diuretic properties show a major advantage compared to thiazides and thiazides-like diuretics, which act on the Na^+^CL^-^ -cotransporter in the distal convoluted tubule, or loop diuretics, which block the Na-K-2Cl co-transporter of both the Henle loop and the macula densa, making the macula insensitive to the increased tubular sodium coming from the Henle loop. In fact, loop diuretics do not activate the TGF and sharply stimulate RAAS. As compared to loop diuretics, SGLT2-i may distinctly regulate the interstitial *versus* intravascular compartment selectively reducing interstitial oedema, which has been observed in HF and DKD, with minimal change in blood volume. Thus, the different volume regulation by SGLT2-I may limit the aberrant neurohumoral and RAAS stimulation that occurs in the setting of volume depletion (54, 55). On the other hand, loop and thiazide/thiazide like diuretics, when used without a RAAS antagonist, do not protect glomeruli from the increased pression and hyperfiltration (41).

Antihypertensive effects of SGLT2-i, which have been reported in clinical trials, are presumably due to their natriuretic effect. SGLT2-i induced natriuresis and glomerular hemodynamics changes resemble, even partially, those observed with sacubitril/valsartan, a first in-class angiotensin receptor neprilysin inhibitor (ARNi). By inhibiting the NP (natriuretic peptide) system, sacubitril/valsartan increases natriuretic peptides (NPs) levels that, in turn, lead to vasodilation, natriuresis and RAAS inhibition through intracellular cyclic guanosine monophosphate (cGMP)-dependent pathways. Both SGLT2-i and valsartan/sacubitril reduce Na+ reabsorption in the proximal tubule and therefore stimulate TGF. Conversely, sacubitril/valsartan has been shown to increase PRA, ANG I and ANG II levels in a canine model of RAAS activation. Furthermore, it significantly decreased aldosterone concentrations, a known predictor of increased cardiovascular mortality risk, which would indicate AT1R blockade (56, 57).

Finally, the prevalence of primary aldosteronism (PA), the most common form of secondary hypertension, is reported to be 11-14% in patients with diabetes and hypertension. PA is also associated with impaired glucose tolerance (IGT). Aldosterone/renin ratio (ARR) is commonly used to screen for PA. Thus, the elucidation of whether the SGLT2-i diuretic effect can influence ARR values for PA screening in patients with diabetes and hypertension is an important clinical issue.

Emerging studies provided evidence that SGLT2-i might have an interfering effect on ARR. Decreasing extracellular fluid volume, they might decrease ARR like thiazide diuretics, leading to false-negative screening for PA in patients with T2DM (58). However, unlike thiazide and loop diuretics, SGLT2-i are not associated to systemic sympathetic hyperactivity, typically found in diabetes. The long-term sympathoinhibition suppresses renin release, mimicking the effects of beta beta-blockers through ß-1 receptors in the renal iuxtaglomerular apparatus, leading to an increase of the ARR.

## Effects of GLP-1 Receptor Agonists on RAAS

GLP-1 receptor agonists (GLP1-RA) are a relatively novel class of anti-diabetic drugs that improve glycemic control by potentiating the physiological effect of the gut hormone GLP-1 (59). GLP-1 indeed stimulates postprandial insulin secretion, inhibits glucagon release, and reduces food intake through the delay of gastric emptying and the suppression of appetite (59). Published trials showed that the treatment with GLP1-RA reduced the risk of MACE and slowed the development of albuminuria in diabetic patients, demonstrating a direct cardio and reno-protective action of GLP1-RA, regardless the effect on glycemic control (60, 61).

Since cardiovascular complications of diabetes (62, 63) as well as diabetic nephropathy (64) are associated with an imbalance in the activity of RAAS, the interaction with this system could explain some of the beneficial effects of GLP1-RA. There is indeed a strong evidence that GLP1-RA counteracts ANG II action at different levels.

Both acute GLP-1 infusion (65, 66) and a single liraglutide dose (67) decreased plasma ANG II concentration in healthy subjects and type 2 diabetic patients, consistently with an inhibitory effect of GLP-1 on renin secretion (68, 69). However, the underlying mechanism has not been fully elucidated. GLP-1 may decrease sodium reabsorption, thus increasing NaCl delivery to the macula densa. The consequent activation of the tubuloglomerular feedback could inhibit renin secretion (70). Alternatively, GLP-1 may act directly on the renin-secreting cells of the juxtaglomerular apparatus, where the expression of GLP-1 receptors has been detected (69). Moreover, GLP-1 could down-regulate ANG II production at tissue level (37). Martins et al. (71) indeed reported that chronic treatment with GLP-1RA exendin 4 (Ex4) in rats decreased renal cortical ANG II content and urinary excretion of AGT, while blockade of GLP-1 receptor with exendin 9 (Ex9) exerted opposite effects. Treatment with GLP1RA also increased the expression and the activity of ACE2 in organs like lungs (20, 72), heart (73, 74), and liver (75), restoring the ACE/ACE2 balance, which is impaired in kidney disease (76), diabetes mellitus (20), and cardiac fibrosis (73); moreover, it has been reported that GLP-1RA down-regulated AT1 and up-regulated AT2 in cardiomyocytes, glomerular capillaries and proximal tubules of the renal cortex in rats (73, 74, 77), resulting in a protective effect against ANG II-induced cardiac and kidney fibrosis (73, 74, 77).

GLP-1 may also inhibit ANG II action at post-receptor level. Several signaling pathways in response to ANG II are mediated by reactive oxygen species (ROS) (78), whose production is increased by ANG II in different cell types through Nox4 (NADPH [Nicotinamide adenine dinucleotide phosphate] oxidase 4) stimulation (21). The increase in oxidative stress is implicated in many pathological conditions associated with RAAS hyperactivation, such as endothelial dysfunction (79), cardiac hypertrophy (80), and diabetic nephropathy (81). GLP-1 receptor stimulation may prevent the increase in ROS induced by ANG II. Ishibashi et al. (82) showed that *in vitro* GLP-1 inhibited the ANG II-induced mesangial cell damage by suppressing superoxide-mediated nuclear factor-kB activation. Similarly, Okabe et al. (80) reported that treatment with teneligliptin, a dipeptidyl peptidase (DPP)–4 inhibitor, suppressed ANG II-induced increase in Nox4 mRNA in rat cardiomyocytes, thus attenuating the ANG II-induced cardiac hypertrophy. The antioxidant action of GLP-1 is mediated by the activation of the cyclic AMP (cAMP) - protein kinase A (PKA) pathway, which downregulates the activity of Nox4 (82, 83). Moreover, Mima et al. (84) showed that the PKA pathway also mediated the GLP-1-induced inhibition of ANG II signaling on cRAF (Ser 259) in glomerular endothelial cells, providing a further explanation for the renal protective effects of GLP-1.

**Figure 2:**
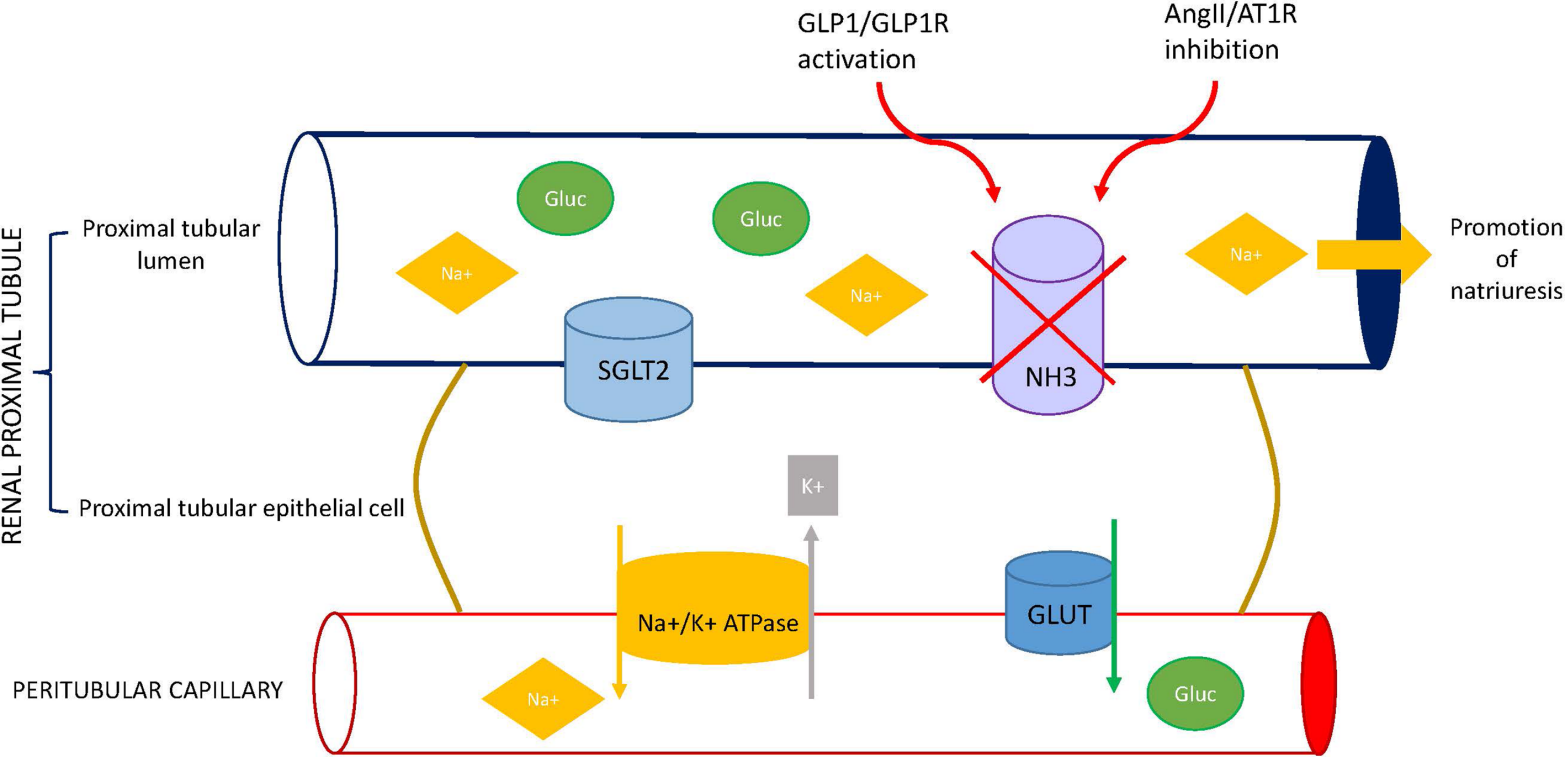
Effect of GLP1 receptor agonists (GLP1-RA) on sodium balance. GLP-1R, glucagon-like peptide-1 receptor; ANG II, angiotensin II; AT1R, angiotensin II type 1 receptor; SGLT2, sodium-glucose cotransporter 2; NHE3, Na+/H+ exchanger isoform 3; GLUT, glucose transporter.

Finally, GLP-1 and ANG II interact in the regulation of sodium and water balance at proximal tubular apical membrane. The tubular proximal reabsorption of sodium is mainly mediated by the Na^+^/H^+^ exchanger isoform 3 (NHE3), a protein located in the brush-border epithelium of the proximal tubule (85). ANG II stimulates NHE3 activity, increasing proximal tubule sodium and water reabsorption (86, 87). Conversely, both acute GLP-1 infusion (65, 70) and a single exenatide injection (88) induced natriuresis in healthy and obese subjects. The natriuretic action of GLP-1 is mainly mediated by inhibition of NHE3 activity through a PKA-dependent mechanism (71, 89–91) (**Figure 2**).

Although data from literature indicate that GLP-1 inhibits ANG II action, its effect on aldosterone is less established. Baretic et al. (92) and more recently Heinla et al. (68) reported that a single GLP-1 infusion decreased aldosterone levels in healthy, normal weight subjects. Conversely, both Skov et al. (65) and Asmar et al. (66) did not find changes in aldosterone concentrations under comparable conditions. However, aldosterone secretion is regulated by several factors besides ANG II, including potassium concentration, ACTH, and membrane depolarization of zona glomerulosa cells (93). Thus, variations of each single factors could potentially explain the discrepancies observed in the different studies (66). Moreover, short-term experiments may not be able to detect the impact of ANG II suppression on aldosterone levels, since aldosterone has slower rates of secretion and degradation than ANG II (66). The chronic effect of GLP1-RA on adrenal function was investigated by Sedman et al. (94), who reported increased aldosterone and renin concentrations in healthy subjects after three weeks of treatment with liraglutide. However, the finding was considered secondary to the reduction of blood pressure rather than a direct effect of the drug on the RAAS.

## Conclusion

In conclusion, the emerging observation that some complications of diabetes are associated with an hyperactivation of RAAS has provided the pathophysiological basis to study the effects of GLP1-RA and SGLT2-i on RAAS to better explain the cardio-reno-protective effect demonstrated by the clinical trials, beyond their role on glucose control. Although underlying mechanisms are still not completely explained, the interaction with RAAS seems to play a relevant role. Interestingly, the influence on RAAS should also be considered in the clinical practice during the screening of suspected hyperaldosteronism due to the interfering regulation of sodium and water balance or mimicking the effects of beta-blockers (SGLT2-i).

## Author Contributions

All authors contributed to the article and approved the submitted version.

## Funding

The research has been supported by funding from Ricerca Locale Università di Torino 2020 — RILO 2020.

## Conflict of Interest

The authors declare that the research was conducted in the absence of any commercial or financial relationships that could be construed as a potential conflict of interest.

## Publisher’s Note

All claims expressed in this article are solely those of the authors and do not necessarily represent those of their affiliated organizations, or those of the publisher, the editors and the reviewers. Any product that may be evaluated in this article, or claim that may be made by its manufacturer, is not guaranteed or endorsed by the publisher.
